# Factors perceived to influence risky sexual behaviours among university students in the United Kingdom: a qualitative telephone interview study

**DOI:** 10.1186/1471-2458-14-1055

**Published:** 2014-10-09

**Authors:** Elton Chanakira, Alicia O’Cathain, Elizabeth C Goyder, Jennifer V Freeman

**Affiliations:** School of Health and Related Research, University of Sheffield, Sheffield, UK

**Keywords:** Young people, University students, Sexual behaviour, Sexually transmitted infections, Public health, Qualitative research

## Abstract

**Background:**

In the United Kingdom people under the age of 25 years are at increased risk of contracting sexually transmitted infections. Most university students belong to this age group but little is known about their sexual behaviours. The aim of the study was to explore university students’ perspectives of factors and mechanisms that influence risky sexual behaviours among university students in the United Kingdom.

**Methods:**

All students at a university in a northern city of England were invited via email to participate in qualitative telephone interviews. Interviews were audio recorded and transcribed verbatim. Framework analytical approach was used.

**Results:**

Twenty interviews were conducted with a diverse sample of students. The social context of university lifestyle was perceived to affect risky sex through high levels of alcohol consumption, increased sexual opportunities, liberation from moral surveillance and expectations of the stereotypical highly sexually active student. Individual and cultural differences were also perceived to account for some patterns of risky sex with older students, overseas students and religious students perceived to be less likely to engage in risky sex due to academic priorities and a tendency to be more likely to adhere to moral values. Risk denial was also a key factor that led students to engage in risky sex. Poor access to sexual health services including inconvenient opening times, lack of confidentiality and stigma were perceived to contribute to the limited use of sexually transmitted infections testing and contraceptive services.

**Conclusions:**

Lifestyle, individual and structural factors seem to play an important role in influencing the risky sexual behaviours of university students. Therefore preventive interventions that focus on these factors could be very useful in this sub-population of young people. This study provides useful baseline information that helps us understand how and why some United Kingdom university students engage in risky sexual behaviours that puts them at risk of contracting sexually transmitted infections.

## Background

In England and the United Kingdom (UK) high rates of the most commonly diagnosed sexually transmitted infections (STIs) tend to be observed among young people under 25 years old [1, 2]. For instance, the UK prevalence rates for chlamydia are highest among 18–19 year old women (4.7%) and men aged 20–24 years (3.4%) and for human papillomavirus (high-risk types) women aged 18 – 19 years have the highest rates (29.6%) followed by those in the 20 – 24 year age group (26.6%) [3]. These high rates of infection are mainly due to risky sexual lifestyles (such as having intercourse without a condom, having multiple sexual partners and having one night stands – referred to as casual sex in this paper) of these age groups [4–6]. Most UK university students belong to these age groups, but there is limited literature about the risky sexual behaviours of this sub-group of young people.

Sexual behaviour is complex partly because it is influenced by a wide array of personal, social, cultural, moral and legal factors [5, 7, 8]. This underscores the importance of understanding the behaviours of specific population groups. Most of the qualitative studies that have investigated students’ sexual behaviour in the UK tend to target young people at schools, colleges, clinics, community and youth centres [9–15]. Although some studies have included university students [16, 17], these studies tend to have no subgroup analysis of university students making it difficult to draw conclusions relevant to this group.

University students in the UK are an important but often neglected public health population group [18]. The reasons for lack of research on this population is unclear; but it is suggested that poor response rates, academic and social distractions [18] and the presumption that university students are “well educated and health conscious” [19] may help explain this paucity.

There is little information about factors that influence risky sex among UK university students and our study sought to gain university students’ perspectives of factors and mechanisms that shape the risky sexual behaviours that put university students in the UK at risk of contracting STIs.

## Methods

### Design

We undertook this qualitative research as part of a larger mixed methods research project that investigated the sexual lifestyles of university students in the UK. Qualitative research was chosen for this part because it is useful in situations where there is little pre-existing knowledge, the issues are complex and the maximum opportunity for exploration is desired [20]. For this part of the study, semi-structured telephone interviews were used because the relative anonymity offered by this mode tends to reduce the embarrassment involved in responding to socially loaded questions compared to face to face situations [21].

### Recruitment and participant selection

In December 2008, all students registered at a university in the north of England were sent an email inviting them to voluntarily take part in the study. A hypertext link to a dedicated study website was embedded in the invitation email. The website contained detailed information about the study including an information sheet and an interview topic guide.

Approximately 24,000 students were registered that year and 273 volunteered to participate. With such a large pool of students expressing an interest in participating, a procedure similar to a random purposeful sampling strategy was used to select participants [22]. This was done by classifying all cases with similar characteristics in terms of gender (male/female), age group (18–19; 20–24; 25–34; 35–44; over 45), level of study (undergraduate, postgraduate taught and research) and year of study (1^st^; 2^nd^; 3^rd^; 4^th^). Fourteen groups were identified and all cases in each group were then assigned a number and were drawn in turn using a computer generated random number generator.

### Data collection procedures

All participants were informed that the interviews would be audio recorded and they were asked to reaffirm their consent. A field diary was kept to record key issues and reflections of the interviews. All interviews were conducted by E. Chanakira (EC) who was a public health PhD trainee at the time. During data collection, A. O’Cathain (AO) a Professor of Health Services Research with extensive qualitative experience provided close supervision and advice on interviewing technique. Twenty interviews were conducted by telephone in two phases; the first ten were conducted in December 2008 and the second phase was undertaken between April and June 2009. The main purpose of conducting the interviews in two phases was to allow preliminary analysis of the data to identify key areas for further exploration and consider data saturation. During the second phase of interviews greater attention was paid to the emergence of new issues and insights. After twenty interviews issues already identified earlier were being repeated and a decision was made to stop interviewing. The decision to stop interviewing was also partly guided by our preliminary survey analysis (some of these findings have been published elsewhere [23]), which initially showed similar factors to be related to risky sexual behaviour among students.

All participants were offered £10 cash to compensate them for their time. The study was approved by the University of Sheffield Ethics Committee.

### Analysis

All interviews were transcribed verbatim and framework analytical approach was used [24]. Framework was considered appropriate because it allows for themes to be based on a priori issues and themes can also emerge from the interview data itself.

Framework analysis involves the following interconnected stages: familiarisation, identifying a thematic framework, indexing, charting and, mapping and interpretation [24, 25]. Familiarisation involved immersion in the data to gain a thorough overview of the material collected. The thematic framework was identified by using existing literature about young people’s sexual behaviour and the preliminary results of the sexual lifestyle survey which was part of our wider study. Emergent themes were also identified from field diary notes and the interviews themselves. Indexing involved reading and annotating all transcripts by hand according to the thematic framework using a textual system. To enhance credibility, AO indexed one transcript and this was compared with EC index codes for the same interview and no major indexing differences were found. Charting involved lifting data from their original context and rearranging distilled summaries of views and experiences of all participants into appropriate thematic reference. Mapping and interpretation involves pulling together the data set as whole to determine meaning, salience and connections of key issues [24, 25]. Although most of the analysis was conducted by EC, all authors discussed the development of the thematic framework and interpretation of findings. This manuscript adheres to RATS guidelines for reporting qualitative research studies.

## Results

Twenty two volunteers were approached for interview; one of these could not participate because they were admitted in hospital and another student could no longer participate because they were busy. Twenty students finally participated, of which participants were equally distributed by gender and half were under 25 years old (Table 1). The interviews were typically an hour long. The major themes identified are described below.

**Table 1:** **Characteristics of participants**

ID	Gender	Age group	Level of study	Year of study
1	Female	18 - 19	Undergraduate	1^st^
2	Male	Over 45	Postgraduate Research	3^rd^
3	Female	20 - 24	Postgraduate Taught	1^st^
4	Male	20 – 24	Postgraduate Research	3^rd^
5	Female	25 – 34	Postgraduate Taught	1^st^
6	Female	25 – 34	Undergraduate	1^st^
7	Male	25 – 34	Postgraduate Taught	2^nd^
8	Male	20 – 24	Postgraduate Taught	1^st^
9	Female	20 - 24	Undergraduate	1^st^
10	Female	20 – 24	Undergraduate	5^th^
11	Female	20 - 24	Postgraduate Taught	1^st^
12	Female	Over 45	Postgraduate Taught	1^st^
13	Male	20 – 24	Postgraduate Taught	1^st^
14	Male	25 - 34	Postgraduate Research	1^st^
15	Female	35 – 44	Postgraduate Research	4^th^
16	Male	25 – 34	Postgraduate Taught	1^st^
17	Male	18 – 19	Undergraduate	2^nd^
18	Male	20 – 24	Undergraduate	3^rd^
19	Male	25 – 34	Undergraduate	5^th^
20	Female	25 – 34	Undergraduate	2^nd^

### University lifestyle

Almost all participants spontaneously viewed alcohol as the cornerstone of students’ social life and it was viewed as playing a key role in facilitating sexual encounters. In particular participants were of the view that the disinhibition effect of alcohol and the strategic use of alcohol to facilitate sexual encounters were major contributors of students engaging in risky activities such as unprotected sex and casual sex.

*“I think people can make a conscious decision to get a bit out of [their] head…on drink and [hope that they are] going to get laid*…*” (****20****)*

Participants also indicated that a university environment increased the opportunity for sexual encounters compared to other social contexts because of the large number of young people of similar age living in the same location.

*“… but you know [students] are all [driven] by the fact that they have easy access to sex…this kind of environment gives them more access…” (****5****)*

The predominant view within the sample was that this opportunity increased in the first year of study but it diminished in subsequent academic years. The only exception was the first few weeks of the beginning of each academic year, when those who had been at university the previous year were perceived to target new students as potential sexual conquests because new students were perceived to be sexually naïve. Most participants were also of the view that the longer students were at university the more their priorities change from social thrills to academic performance. It was implied that having more academic priorities reduced the likelihood of leading a risky sexual lifestyle. However, one postgraduate participant pointed out that the key issue was sexual opportunities rather than differing priorities.

*“…it’s just basically being a [postgraduate] kind of limit your access that’s all otherwise we will be running around just like the undergraduates…” (****5****)*

Participants identified independent living, increased sexual opportunities and social expectations as contributing to a risky sexual lifestyle for students. For example, it was thought that the sense of liberation from parental and community moral surveillance increased sexual opportunities, risky sexual practices and other risky behaviours such as alcohol misuse. Several participants implied that students were not worried about social censure from other students partly because sexual risk was viewed as part of a normative university lifestyle.

*“…you have not got a moral authority to bow down to anymore…certainly in terms of your [parents, siblings, relatives or your old collection of friends] …” (****11****)*

Participants also thought that most students when they come to university were expected to have a very active social life which often included partying, drinking alcohol and being highly sexually active. The latter was widely believed to exert intense social pressure for students to conform to the student stereotype. As one male student puts it,

*“…so there is this burden on people to lose it [virginity] while they are at university… [and if you] have not lost it then you have this huge thing to lose” (****4****)*

The media was considered to play a crucial role in shaping expectations by portraying students as sexually reckless. These sexual representations were considered important because some students would attempt to replicate these images in their real life behaviour. This suggests that some students are merely social actors who engage in risky sex to conform to the perceived risky student lifestyle.

### Individual and cultural differences

Despite the apparent strong influence of university lifestyle, participants also indicated that individual and cultural background differences may help explain the risky sexual behaviours of some students. For instance religion was considered very influential with most participants of the view that it provided a moral framework that sanctioned safe sexual behaviour like abstinence.

*“I think most religions have a strict sort of guidance on whether or not you shouldn’t be having sex before marriage…and they are less likely to break that rule…even under the influence of university thingy…” (****10****)*

Country of origin was also thought to influence sexual behaviour through other socio-cultural factors such as alcohol, religion and attitudes towards sex. For instance, it was argued that students from Western countries were more likely to engage in risky sex compared with those from other parts of the world because of permissive attitudes towards risky sex.

*“…the culture here [UK] is also supportive for this type of behaviours [risky sex]…which is not really possible in some other areas of the world…” (****14****)*

Some participants suggested that due to social expectations male students tend to exaggerate their risky sexual experiences than females. However, in terms of actual behaviour the predominant view was that gender seemed to play a less important role in shaping risky sex because at university females tend to behave in similar ways as their male counterparts; a social phenomenon described by one male participant as *“the ladette culture”****(16)*** – the idea that young women can behave like men in terms of risky behaviours such as binge drinking, casual sex and having multiple sexual partners. It was also suggested that females and males at university experience the same emotional rewards and penalties for their sexual behaviour.

*“The typical view of guys is that they are like boasting to their guy friends about what they have done but there is certain amount of exaggeration in that I am sure” (****1****)*

“*I used to think it was more the boys worse than the girls [being promiscuous] but…I don’t really see a difference nowadays. I think they are all quite similar in what they after” (****6****)*

“…*it’s pretty similar between men and women…from my experience, I would say it’s probably not that much different at university, but [as] I said perhaps less so at university than in other parts of life… (****10****)*

“…*I don’t really feel that there is that much of a dividing line between how boys act and how girls act. I don’t think you can really say right boys are out to get as much as possible and girls are like, they just wait until they get drunk and are taken advantage of. I think it works both ways…I think there are some girls who are just as promiscuous as boys and feel just as good about it and I think that guys sometimes go out and sleep with people and feel slutty as well”. (****11****)*

In contrast, age was considered influential in students’ risky sexual behaviour. Participants suggested that differences in sexual risk were explained by a complex mix of biological and social mechanisms. For example, it was thought younger students were biologically highly sexually active, therefore more likely to have multiple sexual partners. On the other hand, sexual risk taking was also considered to be socially embedded in the culture of young people and university lifestyle.

*“…people of that age…are more sexually active anyway because that’s just the way the body work…and the libido drops off with age…” (****9****)*

*“…I think there is a general culture around the university that this is the time, this is the age [when] we are going to be experimenting…” (****12****)*

Risk denial was also identified as another key individual factor that influenced risky sex. As one postgraduate female puts it, *“…the ones that think it will never happen to them are the ones that don’t use condoms; they are the ones that sleep with as many people as they want” (****3****)*. The reasons for risk denial were thought to be complex and varied. Some participants suggested that even with good knowledge and STI awareness, some students simply ignore this knowledge or they tend to act impulsively because the short term benefits of sexual thrills far outweighed facing up to the potential negative costs of risky sexual encounters.

*“…I don’t think [students] think about [sexual risks] enough…they’re just thinking about that moment and [they just want to have] a quickie …” (****12****)*

Several other reasons were given to explain why students might be in risk denial. A few participants speculated that most students might feel that STIs usually affect people of low social class and those that are less educated. It was also suggested that students were more concerned about reducing the risk of pregnancy rather than STIs.

### Poor access to sexual health services

Interviewees were asked their views about the low uptake of STI testing among students as demonstrated by participants in the main sexual lifestyle survey that this current study was designed to complement. The predominant view was that students in general were unable to access sexual health services for STI screening, treatment, contraception supplies and advise because these services were open at inconvenient times. Concerns about confidentiality and the stigma associated with accessing sexual health services were also considered major barriers when accessing dedicated sexual health services.

*“…I know that the Family Planning Clinic isn’t open all the time and for all the [students who are] busy at Uni. [they] haven’t got [the] time to go to that clinic…” (****9****)*

*“You go to the GUM clinic…and it’s pretty visible the way you walk in… perhaps just walking in would be a bit of a deterrent… [and] going to a doctor [and say]…I am worried that I may have a sexually transmitted infection; [students]…may be worried about the response they get from the doctor or nurse” (****10****)*

## Discussion

This study explored university students’ views about factors that influenced risky sexual behaviours among university students. Our findings suggest that in this group a wide array of lifestyle, cultural, individual and structural factors influence risky sexual behaviours.

The combination of alcohol use, the freedom associated with living away from home and the social expectations of a risky student sexual lifestyle seem to play a central role in shaping the risky sexual lifestyle of university students. However, gender sex role stereotypes did not seem to shape expectations and risky sexual behaviour of students and this is consistent with evidence showing gender similarities in sexual behaviour and attitudes [26]. However, others have observed a contrary pattern among other young people worldwide [27] and in the UK [11].

In particular, alcohol consumption was viewed as a social lubricant at the centre of most students’ social life and our findings suggests its use in social contexts often led to risky sexual behaviour. This is consistent with findings from other studies of young people in the UK [9, 17, 28, 29] and university students in other countries [30, 31] showing that alcohol is perceived to influence risky sex.

Living independently away from home was considered important because it facilitated students to have sex with many different partners without fear of social censure from peers or community members. This implies that living a risky sexual lifestyle is socially accepted at university and this is consistent with the view that young people often look to their social contexts for clues about what constitutes acceptable sexual behaviour [32]. Our findings are also similar to university students in the UK and other countries [29, 33], and young people undertaking temporary work at large UK holiday centres [17].

Our findings also suggest that students engage in risky sexual behaviours because they perceive themselves as a low STI risk group when they compare themselves to other population groups. This is contrary to the perceptions of other groups of young people, who tend to perceive university students as the group at greatest risk of STIs [16]. This suggests that the existence of optimistic bias the idea that one is less at risk when compared to others [34] cannot be discounted for both, young people in general and university students in the UK. Optimistic bias is important because it is thought to affect STI risk reduction motivations [34].

Our findings also indicate that some students do not have easy access to sexual health services. For example, it was suggested that most specialist sexual health services are open at inconvenient times. Despite legal provisions ensuring the confidentiality of those accessing sexual health services in the UK, evidence from our study suggests that these services are still perceived to be inadequately confidential and stigmatised. These issues have been found to be key barriers of sexual health utilisation by other young people in the UK [11, 16, 35, 36]. To improve access to sexual health services these perceptions need to be addressed and sexual health service providers should also consider having flexible services that are open when most students are less likely to have academic and social commitments.

The lack of visual contact with participants did not seem to compromise the quality of our data. For example, there was great rapport between interviewer and participants, with most interviews typically an hour long and key issues were discussed uninhibited and in-depth. These methodological strengths are consistent with others who argue that qualitative telephone interviews are just as good as face to face interviews [21, 37]. Additionally telephone interviews have many advantages, including ensuring participant anonymity, accessing hard to reach groups and reduced costs [21, 37–39].

Although our sample was diverse, a limitation was that we had fewer younger students (especially those in the 18 – 19 year age group) and slightly more postgraduates and this may limit the transferability of our findings. Transferability refers to the degree to which the results of qualitative research can be transferred to other contexts or settings and this has been proposed as a more appropriate alternative to generalizability which is often used in quantitative research [40]. As for our findings, having slightly older young students and postgraduates was arguably advantageous because these groups could reflect on their younger colleagues and their own past experience in their undergraduate years.

Our findings are also potentially incomplete mainly because we cannot guarantee that we absolutely attained data saturation, because as argued by others [41] the longer one examine and analyse their data there will always be the potential for new data to emerge. However, it should be acknowledged that the point of data saturation tends to be difficult to identify [42]. In our study we made a decision to stop further data collection after twenty interviews because it appeared that no new themes were emerging during the second phase of our data collection. Although guidelines for sample size in qualitative research are varied and debatable [42], it is acknowledged that qualitative research typically involves the intensive study of a small group of people and tends to focus on depth rather than breadth [43].

Our sample size also need to be viewed in the context that our qualitative study was not designed to be representative of students’ views but it was designed to complement our quantitative survey. That is, our qualitative study was designed to provide an explanation of factors that were identified in our survey as contributing to risky sexual behaviours among university students. This is consistent with the view that the main purpose of qualitative research is exploring meaning and contextual uniqueness attributed to a phenomena rather than generalisations [43–45].

The dearth of sexual behaviour literature among UK university students also makes it difficult to compare our findings. Nonetheless, existing data from other university students in other countries and subgroups of young people in the UK seem to corroborate some of our key findings.

## Conclusions

Despite some of the limitations, our study has made an important first step towards understanding the factors and mechanisms specific to UK university students that explain their risky sexual behaviour. Our findings suggest that a dynamic interplay of individual, socio-demographic, lifestyle and structural factors influence risky sexual behaviours among university students. Taken together it would seem that the university social environment inadvertently plays a major part in explaining the risky sexual lifestyles of university students. However, our findings are tentative and require further data to corroborate them. To improve sexual health in this population prevention strategies need to consider lifestyle, individual and improved access to sexual health services.

## References

[1] Public Health England (2013). Sexually transmitted infections and chlamydia screening in England, 2012. Health Protect Rep.

[2] Public Health England (2013). Sexually Transmitted Infections Annual Data.

[3] Sonnenberg P, Clifton S, Beddows S, Field N, Soldan K, Tanton C, Mercer CH, da Silva FC, Alexander S, Copas AJ, Phelps A, Erens B, Prah P, Macdowall W, Wellings K, Ison CA, Johnson AM (2013). Prevalence, risk factors, and uptake of interventions for sexually transmitted infections in Britain: findings from the National Surveys of Sexual Attitudes and Lifestyles (Natsal). Lancet.

[4] Johnson AM, Mercer CH, Erens B, Copas AJ, McManus S, Wellings K, Fenton KA, Korovessis C, Macdowall W, Nanchahal K, Purdon S, Field J (2001). Sexual behaviour in Britain: partnerships, practices, and HIV risk behaviours. Lancet.

[5] National Institute for Health and Clinical Excellence (NICE) (2007). One to one Interventions to Reduce the Transmission of Sexually Transmitted Infections (STIs) Including HIV, and to Reduce the Rate of Under 18 Conceptions, Especially Among Vulnerable and at Risk Groups. NICE Public Health Guidance 3.

[6] Mercer CH, Tanton C, Prah P, Erens B, Sonnenberg P, Clifton S, Macdowall W, Lewis R, Field N, Datta J, Copas AJ, Phelps A, Wellings K, Johnson AM (2013). Changes in sexual attitudes and lifestyles in Britain through the life course and over time: findings from the National Surveys of Sexual Attitudes and Lifestyles (Natsal). Lancet.

[7] Fenton KA, Johnson AM, McManus S, Erens B (2001). Measuring sexual behaviour: methodological challenges in survey research. Sex Transm Infect.

[8] Department of health and cross Government (2013). A Framework for Sexual Health Improvement in England. 18420.

[9] Coleman LM, Cater SM (2005). A qualitative study of the relationship between alcohol consumption and risky sex in adolescents. Arch Sex Behav.

[10] Coleman LM (2001). Young people’s intentions and use of condoms: qualitative findings from a longitudinal study. Health Educ J.

[11] Stanley N (2005). Thrills and spills: young people’s sexual behaviour and attitudes in seaside and rural areas. Health Risk Soc.

[12] Jones RJ, Haynes R (2006). The association between young people’s knowledge of sexually transmitted diseases and their behaviour: a mixed methods study. Health Risk Soc.

[13] Brown S (2012). Young men, sexual health and responsibility for contraception: a qualitative pilot study. J Fam Plann Reprod Health Care.

[14] Say R, Mansour D (2009). Contraceptive choice for young people. J Fam Plann Reprod Health Care.

[15] Smith A (2001). Young people’s contraception and sexual health: report of a local needs assessment in Staveley, North Derbyshire. J Fam Plann Reprod Health Care.

[16] Lorimer K, Reid ME, Hart GJ (2009). “It has to speak to people’s everyday life”: qualitative study of men and women’s willingness to participate in a non-medical approach to Chlamydia trachomatis screening. Sex Transm Infect.

[17] Hennink M (2000). Seasonal work and sexual behaviour - statistical data included. J Sex Res.

[18] Stewart-Brown S, Evans J, Patterson J, Petersen S, Doll H, Balding J, Regis D (2000). The health of students in institutes of higher education: an important and neglected public health problem. J Public Health Med.

[19] Zimmer JC, Thurston WE (1998). Attitudes, beliefs, and practices of nursing students concerning HIV/AIDS: Implications for prevention in women. Health Care Women Int.

[20] Bowling A (2002). Research Methods in Health: Investigating Health and Health Services.

[21] Sturges JE, Hanrahan K (2004). Comparing telephone and face to face qualitative interviewing: a research note. Qual Res.

[22] Sandelowski M (2000). Focus on research methods: combining qualitative and quantitative sampling, data collection, and analysis techniques in mixed method studies. Res Nurs Health.

[23] Chanakira E, Goyder E, Freeman J, O’Cathain A, Kinghorn G, Jakubovic M (2014). Social and psychosocial factors associated with high risk sexual behaviour among university students in the United Kingdom: a web-survey. Int J Std Aids.

[24] Ritchie J, Spencer L, Bryman A, Burgess RG (1994). Qualitative data analysis for applied policy research. Analyzing Qualitative Data.

[25] Ritchie J, Spencer L, O’Connor W, Ritchie J, Lewis J (2003). Carrying out qualitative analysis. Qualitative Research Practice: A Guide for Social Science Students and Researchers.

[26] Petersen JL, Hyde JS (2011). Gender differences in sexual attitudes and behaviors: a review of meta-analytic results and large datasets. J Sex Res.

[27] Marston C, King E (2006). Factors that shape young people’s sexual behaviour: a systematic review. Lancet.

[28] Dale H, Watson L, Adair P, Moy M, Humphris G (2010). The perceived sexual health needs of looked after young people: findings from a qualitative study led through a partnership between public health and health psychology. J Public Health.

[29] Farrow R, Arnold P (2003). Changes in female student sexual behaviour during the transition to university. J Youth Stud.

[30] Gilchrist H, Smith K, Magee CA, Jones S (2012). A hangover and a one-night stand: alcohol and risky sexual behaviour among female students at an Australian University. Youth Stud Aust.

[31] Scott-Sheldon LAJ, Carey MP, Carey KB (2010). Alcohol and risky sexual behaviour among heavy drinking College students. AIDS Behav.

[32] Shoveller JA, Johnson JL, Langille DB, Mitchell T (2004). Socio-cultural influences on young people’s sexual development. Soc Sci Med.

[33] Tura G, Alemseged F, Dejene S (2012). Risky sexual behaviour and predisposing factors among students of Jumma University, Ethiopia. Ethiop J Health Sci.

[34] Weinstein ND (1980). Unrealistic optimism about future life events. Soc Psychol.

[35] Carroll C, Lloyd M, Cooke J, Owen J (2011). Reasons for the use and non-use of school sexual health services: a systematic review of young people’s views. J Public Health.

[36] Baxter S, Blank L, Guillaume L, Squires H, Payne N (2011). Views of contraceptive service delivery to young people in the UK: a systematic review and thematic synthesis. J Fam Plann Reprod Health Care.

[37] Novick G (2008). Is there a bias against telephone interviews in qualitative research?. Res Nurs Health.

[38] Irvine A, Drew P, Sainsbury R (2010). Research Works 2010–03. Mode Effects in Qualitative Interviews: A Comparison of Semi-Structured Face to Face and Telephone Interviews Using Conversation Analysis.

[39] Opdenakker R (2006). Advantages and disadvantages of four interview techniques in qualitative research. Forum.

[40] Lincoln YS, Guba E (1985). Naturalistic Inquiry.

[41] Strauss A, Corbin J (2008). Basics of Qualitative research: Techniques and Procedures for Developing Grounded Theory.

[42] Mason M (2010). Sample size and saturation in PhD studies using qualitative interviews. Forum.

[43] Bryman A (2012). Social Research Methods.

[44] Denzin NK, Lincoln YS (2011). Handbook of Qualitative Research.

[45] Bowling A (2009). Research Methods in Health: Investigating Health and Health Services.

[CR46] The pre-publication history for this paper can be accessed here:http://www.biomedcentral.com/1471-2458/14/1055/prepub

